# SPTNet: SuperPoint Tracking Network for Visual SLAM

**DOI:** 10.3390/s26144612

**Published:** 2026-07-21

**Authors:** Min Pang, Jichao Jiao, Yingjian Zhang

**Affiliations:** 1 School of Electronic Engineering, Beijing University of Posts and Telecommunications, No. 10 Xitucheng Road, Haidian District, Beijing 100876, China; 2 China Research Institute of Radiowave Propagation, No. 33 Xianshan East Road, Chengyang District, Qingdao 266107, China

**Keywords:** deep learning, neural networks, SLAM, feature matching, optical flow

## Abstract

Robust image-to-image correspondence is a fundamental challenge for camera-based Visual Simultaneous Localization and Mapping (SLAM). Conventional approaches primarily rely on isolated local feature matching or optical flow prediction, which often suffer from limited robustness under large parallax, high computational overhead, and cumulative drift errors during long-sequence tracking. To address these limitations, we propose SPTNet (SuperPoint Tracking Network), an efficient multi-task neural network that tightly couples feature detection, description, and dense optical flow prediction within a unified architecture. The fundamental innovation of SPTNet is a Hybrid Tracking Module (HTM) governed by a novel Predictor-Corrector mechanism. Specifically, the dense optical flow field acts as a temporal prior to constrain the descriptor matching search space, while the descriptors act as a correction signal, eliminating flow-induced drift at each frame through spatially constrained Sinkhorn optimization. This synergy enables efficient feature reuse via a shared backbone, minimizing redundant computation. Comprehensive experiments on indoor and outdoor datasets demonstrate that SPTNet attains a false matching rate as low as 1.8% at a 5-pixel threshold on HPatches, substantially reduces cumulative drift on long-sequence SLAM benchmarks, and maintains a high execution speed of 35 FPS on standard GPUs, demonstrating a highly compact footprint advantageous for prospective embedded robotic deployment.

## 1. Introduction

Visual Simultaneous Localization and Mapping (SLAM) has emerged as a foundational cornerstone for autonomous navigation, robotics, and intelligent vehicles, where the primary objective is to estimate camera ego-motion while concurrently constructing a coherent representation of the surrounding environment [1]. Since the seminal dual-thread paradigm established by MonoSLAM [2] and PTAM [3], the visual SLAM pipeline has increasingly transitioned from hand-crafted geometric engineering to deep learning-driven front-ends. In these pipelines, the accuracy and robustness of the front-end tracking module directly govern the fidelity of downstream state estimation and backend factor-graph optimization. Despite substantial progress, long-term, high-fidelity data association remains a formidable challenge. This difficulty is particularly pronounced under extreme viewpoint changes, dynamic lighting, and severe texture scarcity.

In the current landscape of visual front-ends, two predominant paradigms for temporal data association coexist: feature-based matching (both detector-based and detector-free) and predictive optical flow estimation. Feature-based methods, such as modern deep descriptors, are robust against large displacements and appearance changes. However, they incur high latency from global descriptor extraction and exhaustive nearest-neighbor matching. They are highly susceptible to perceptual aliasing, where repetitive environmental patterns induce catastrophic false matches. Conversely, predictive optical flow methods provide dense, pixel-wise temporal continuity at high efficiency. Yet they are inherently open-loop, accumulating integration drift and aperture ambiguity. Without explicit spatial anchors, they cannot self-correct during long-sequence tracking. Regrettably, conventional SLAM architectures treat these two paradigms as isolated, parallel stages, preventing them from mutually correcting their respective failure modes and allowing drift to accumulate unchecked.

To bridge this gap, we propose SPTNet (SuperPoint Tracking Network), a unified, multi-task framework designed to unlock high-efficiency, drift-free feature tracking for real-time visual SLAM. The core insight of SPTNet is that feature localization and instantaneous motion fields should not be merely concatenated, but rather *tightly coupled* in a symbiotic loop. To achieve this, we introduce a novel Predictor-Corrector mechanism within a centralized architecture. Rather than relying on a heuristic multi-head assembly, SPTNet operates a shared lightweight encoder that projects the input stream into a high-dimensional semantic manifold. In this manifold, the optical flow head acts as a temporal “Predictor” to propose correspondences, while the descriptor head serves as a spatial “Corrector” that resets drift via Sinkhorn optimization. This synergy enables efficient feature reuse and achieves robust tracking.

The main contributions of this work are articulated as follows:

- We propose a unified multi-task network, SPTNet, which tightly integrates keypoint detection, discriminative description, and dense motion estimation over a shared feature representation, substantially reducing the computational footprint to satisfy the stringent real-time constraints of embedded robotic platforms.
- We introduce a Hybrid Tracking Module (HTM) governed by a rigorous Predictor-Corrector philosophy, which mathematically bridges the operational gap between instantaneous temporal continuity (flow) and long-term spatial identity (descriptors) to realize drift-free tracking.
- We demonstrate through extensive experimental evaluations on both indoor and outdoor standard benchmarks (including HPatches, KITTI, and TUM RGB-D) that SPTNet achieves state-of-the-art data-association accuracy and tracking robustness, while maintaining low deployment latency suitable for embedded platforms.

**Figure 1:**
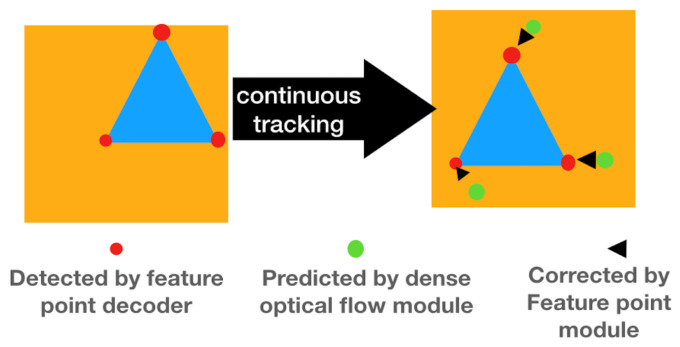
In the continuous tracking process, we use the optical flow module to determine the initial position of the target and subsequently detect feature points to continually refine its position. The consistency of feature point detection on the target’s position enables this approach to accurately track the target.

Figure 1 illustrates this continuous tracking process.

## 2. Related Work

The core of Visual SLAM lies in estimating camera ego-motion and constructing environment representations via consecutive image matching. Since the establishment of the dual-thread tracking and mapping architecture by MonoSLAM [2] and PTAM [3], the field has evolved from handcrafted pipelines to end-to-end frameworks driven by deep neural networks. Recently, research focus has shifted toward achieving robustness under extreme viewpoint variations and dynamic lighting, particularly for autonomous systems with constrained visual sensors. The latest advances point toward generalizable matching [4], powerful 3D foundation models [5,6], and lightweight on-device solutions [7,8]. Despite these strides, a tightly coupled architecture that jointly predicts flow and descriptors in one shot remains underexplored; SPTNet fills this gap.

### 2.1. Local Feature Detection and Matching

Traditional visual SLAM front-ends have long been dominated by hand-crafted feature pipelines such as SIFT [9], SURF [10], and ORB [11]. While computationally efficient, these hand-crafted methods inherently exhibit limited representation capacity under severe illumination fluctuations and viewpoint changes. To mitigate this, deep-learning-driven feature extractors, pioneered by SuperPoint [12] and D2-Net [13], utilize convolutional networks to learn joint keypoint detection and discriminative description. More recent works such as R2D2 [14] further enforce geometric repeatability. To handle complex cross-frame assignments, graph-based matching networks like SuperGlue [15] and detector-free architectures like LoFTR [16] have shifted the paradigm toward global context aggregation via self- and cross-attention mechanisms.

Despite their impressive matching accuracy, these advanced matching networks present two major execution bottlenecks for visual SLAM: first, the exhaustive nearest-neighbor search or attention computation incurs prohibitive latency, violating the strict real-time constraints of embedded robotic platforms; second, when operating in environments with repetitive or homogeneous textures, feature-based paradigms suffer severely from perceptual aliasing, where geometrically distinct areas yield indistinguishable descriptors, resulting in catastrophic tracking failures.

More recently, the community has pushed toward generalizable matching across domains: GIM [4] leverages internet videos for zero-shot transfer, while VGGT [5] and Fast3R [6] treat matching as a byproduct of 3D reconstruction from hundreds of frames. However, these large models are computationally prohibitive for onboard SLAM, where SPTNet offers a lightweight alternative with real-time speed. DeDoDe [17] argues that detection and description may benefit from decoupled training, which is complementary to our work: SPTNet still couples them but mitigates aliasing through the flow-guided predictor, an orthogonal solution.

### 2.2. Deep Optical Flow in Visual SLAM

As an alternative to discrete feature matching, optical flow estimation provides dense, pixel-level temporal continuity by tracking the apparent motion of intensities. Modern learning-based optical flow frameworks have transitioned from early encoder-decoder warping models like PWC-Net [18] to recurrent, all-pairs correlation architectures typified by RAFT [19] and FlowFormer [20]. By maintaining a multi-scale correlation pyramid and iteratively updating the displacement field via Gated Recurrent Units (GRUs), these networks demonstrate remarkable robustness against motion blur and large displacements.

In the context of visual SLAM front-ends, pure optical flow trackers offer high frame-rate motion priors for camera pose initialization without requiring explicit keypoint matching. However, their primary mathematical vulnerability stems from the open-loop nature of continuous integration. Because optical flow merely tracks relative frame-to-frame displacement without maintaining a static spatial identity anchor, it is notoriously plagued by integration drift and aperture ambiguity. Over long-sequence tracking, these subtle frame-level estimation errors accumulate unchecked, leading to massive tracking drift and the eventual corruption of backend factor-graph optimization. Recent end-to-end SLAM systems like DROID-SLAM [21] have attempted to mitigate this by back-propagating through time, but they still lack an explicit descriptor-based correction mechanism.

SEA-RAFT [7] confirms that simplifying RAFT can retain accuracy; SPTNet takes this further by eliminating the separate flow encoder and building the correlation pyramid directly on the shared descriptor feature maps, achieving even greater parameter reduction.

### 2.3. Multi-Task Learning for Visual Front-Ends

To harness the advantages of both geometric stability and temporal continuity, multi-task learning (MTL) has emerged as a promising direction for deep visual front-ends. Frameworks such as OmniGlue [22] or early multi-head networks attempt to output keypoint maps, descriptors, and motion fields concurrently from a centralized backbone. By sharing shallow representation layers, these methods effectively decrease the cumulative computational footprint compared to deploying multiple isolated single-task models.

Regrettably, a critical gap remains in existing MTL front-ends: most architectures merely treat the distinct tasks as parallel, uncoupled heads branching out from a shared encoder. While they achieve computational efficiency at the hardware level, they fail to exploit the algorithmic inter-dependency between tasks during inference. For instance, the predicted motion field is rarely utilized to regularize the descriptor matching domain, and the descriptor matching score is seldom fed back to self-correct the flow field. Consequently, they remain heuristic combinations rather than organic fusions. SPTNet addresses this gap through a tightly coupled predictor-corrector loop, detailed below.

XFeat [8] achieves real-time matching on edge devices through minimal architectures, aligning with our efficiency goal. However, it relies solely on sparse keypoints, whereas SPTNet couples dense flow and sparse descriptors, yielding robustness in textureless regions.

## 3. Methodology

**Figure 2:**
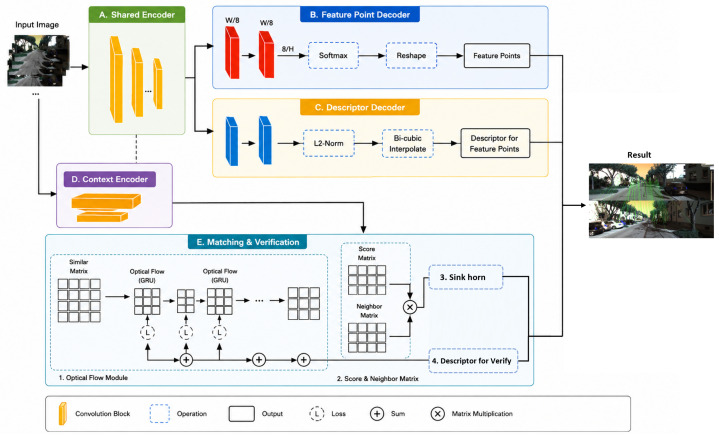
Overview of the proposed SPTNet architecture: (**A**) Shared Encoder; (**B**) Feature Point Decoder; (**C**) Descriptor Decoder; (**D**) Context Encoder; (**E**) Matching & Verification.

**Figure 3:**
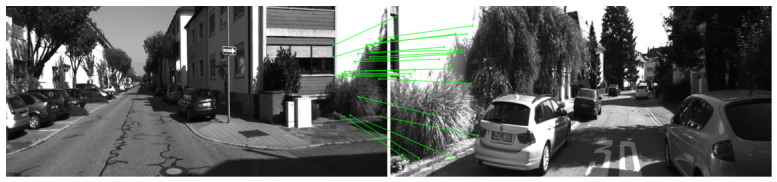
Feature tracking under large parallax. Optical flow (arrows) provides initial displacement estimates, and descriptor matching refines the correspondence within a local search window. This “predict-then-verify” strategy reduces false matches compared to global re-detection.

When designing a joint architecture for feature tracking, two critical observations emerge from the literature: (1) Feature-based detectors and descriptors provide stable, repeatable spatial anchors, yet maintaining consistent detection across large parallax and under motion blur remains highly challenging. (2) Optical flow methods excel at dense temporal continuity and can track a point without explicit re-detection, but they are inherently open-loop, suffering from unbounded integration drift and lacking the reusable descriptors required for map building. To achieve this, we build upon SuperPoint [12] and augment it with a dense optical flow predictor and a feature tracking module. Standard keypoint descriptors, including SuperPoint, tend to fail in textureless areas and under severe motion blur, motivating the integration of dense optical flow as a complementary temporal prior. It can be summarized as (A) Shared Encoder; (B) Feature Point Decoder; (C) Descriptor Decoder; (D) Dense Optical Flow Prediction; (E) Feature Point Tracking Module. The structure is given in Figure 2, and the benefit of feature tracking under large parallax is shown in Figure 3.

### 3.1. System Overview

SPTNet aims to establish robust and temporally consistent feature correspondences between consecutive frames $I_{t}$ and $I_{t+1}$. As illustrated in Figure 2, the network processes input images through a shared encoder to extract high-level semantic features, which are then branched into three specialized heads: the Keypoint Detector, the Descriptor Encoder, and the Dense Optical Flow Predictor. Unlike traditional pipelines that treat these tasks in isolation, SPTNet employs a tightly coupled predictor-corrector strategy.

### 3.2. Multi-Task Network Architecture

Shared Encoder: To ensure real-time performance on embedded platforms, a VGG-style encoder [23] (i.e., an architecture composed of stacked 3×3 convolutions, following the design pattern of the VGG network family) is employed to downsample the input image to a resolution of $H_{c}=H/8,W_{c}=W/8$. It outputs a 256-dimensional feature map that serves as a universal representation for all subsequent tasks, enabling zero-overhead computational reuse. The encoder comprises 8 convolutional layers with 3×3 kernels (stride 1, padding 1), with channel dimensions of 64, 64, 64, 64, 128, 128, 256, 256. Max-pooling (2×2, stride 2) is applied after layers 2, 4, and 6, achieving a total downsampling factor of 8.

Feature Point Decoder: Following the architecture of SuperPoint [12], the detection head produces a tensor $X\in R^{H_{c}\times W_{c}\times 65}$. The 65 channels represent local non-overlapping 8×8 grid cells plus an extra “no interest point” dustbin. After applying a channel-wise softmax and removing the dustbin, the tensor is reshaped to the original image dimensions $R^{H\times W}$, yielding a sparse keypoint saliency map. Keypoints are extracted via non-maximum suppression with a 3×3 window and a confidence threshold of 0.015, capped at a maximum of 2000 per frame.

Descriptor Decoder: The descriptor head computes a dense feature map $g_{\theta }\left(I\right)\in R^{H_{c}\times W_{c}\times 256}$ containing $L_{2}$-normalized fixed-length descriptors. For sub-pixel precision in SLAM, bilinear interpolation is applied to provide per-pixel descriptors at the full image resolution.

Dense Optical Flow Module: Our flow module is inspired by RAFT [19], but with a fundamental architectural shift: the correlation layer operates directly on the dense descriptor feature maps $g_{\theta }\left(I\right)$ rather than shallow CNN features. This ensures that motion estimation is guided by invariant semantic information.

1. Correlation Pyramid: For two descriptor maps from $I_{1}$ and $I_{2}$, a 4D correlation volume is constructed:
  (1) $$C_{ijkl}=\sum_{h}g_{\theta }{\left(I_{1}\right)}_{ijh}\cdot g_{\theta }{\left(I_{2}\right)}_{klh}$$
  The volume is pooled to generate multi-scale pyramids $\left\{C^{1},C^{2},C^{3},C^{4}\right\}$, capturing both local precision and global motion context.
2. Iterative Update Operator: A GRU-based update operator iteratively refines the flow estimate $f_{k}$. By retrieving features from the correlation pyramid and concatenating them with context features, the GRU predicts a flow increment that updates the field toward the ground truth.

### 3.3. Hybrid Tracking Module (HTM)

The Hybrid Tracking Module (HTM) implements the core predictor-corrector mechanism to achieve drift-free feature data association across temporal sequences. Unlike the classical Lucas–Kanade tracker, which iteratively minimizes photometric error within a local intensity window, our predictor–corrector loop operates in learned feature and descriptor spaces, enabling robustness to appearance changes and globally consistent matching through Sinkhorn-based optimal transport.

#### 3.3.1. Step A: Flow-Guided Search Constraint

Let $A={\left\{p_{t}^{i}\right\}}_{i=1}^{M}$ denote the set of *M* keypoints detected in frame $I_{t}$, where $p_{t}^{i}={\left[x_{t}^{i},y_{t}^{i}\right]}^{T}$ represents the 2D pixel coordinates. For each keypoint $p_{t}^{i}\in A$, its coarse estimated position ${\hat{p}}_{t+1}^{i}$ in the subsequent frame $I_{t+1}$ is projected via the dense optical flow field f:

(2) $${\hat{p}}_{t+1}^{i}=p_{t}^{i}+f\left(p_{t}^{i}\right)$$

To suppress perceptual aliasing and prune the correspondence search space, we define a spatial constraint mask $B\in {\left\{0,1\right\}}^{M\times N}$, where *N* is the number of keypoints in the candidate set $B={\left\{p_{t+1}^{j}\right\}}_{j=1}^{N}$ extracted from frame $I_{t+1}$. The elements of the binary mask B are rigorously formulated based on the $\ell_{2}$ distance between the projected prior and the candidate detections:

(3) $$B_{i,j}=\left\{\begin{array}{cc} 1, & \mathrm{if}\;∥{\hat{p}}_{t+1}^{i}-p_{t+1}^{j}{∥}_{2}<R_{\mathrm{th}}\\ 0, & \mathrm{otherwise} \end{array}\right.$$

where $R_{\mathrm{th}}$ denotes the localized search radius to avoid notation conflict with network parameters θ.

#### 3.3.2. Step B: Spatially-Constrained Sinkhorn Optimization

Utilizing the dense descriptor maps $g_{\theta }\left(I_{t}\right)$ and $g_{\theta }\left(I_{t+1}\right)$, we construct a raw similarity score matrix $S\in R^{M\times N}$. For each keypoint, its descriptor is obtained by bilinearly interpolating the dense descriptor map $g_{\theta }\left(I\right)$ at the keypoint’s sub-pixel location. The score entry $S_{i,j}$ represents the inner product of the $L_{2}$-normalized descriptors associated with the respective keypoints:

(4) $$S_{i,j}=\langle g_{\theta }\left(I_{t}\right)\left(p_{t}^{i}\right),g_{\theta }\left(I_{t+1}\right)\left(p_{t+1}^{j}\right)\rangle$$

To completely suppress masked-out pairings, we assign −∞ logits before the exponential:

(5) $$L_{i,j}=\left\{\begin{array}{cc} \tau \cdot S_{i,j}, & \mathrm{if}\;B_{i,j}=1\\ -\infty , & \mathrm{otherwise} \end{array}\right.$$

and define $W_{i,j}=\exp\left(L_{i,j}\right)$ (with exp(−∞)=0). These zero entries remain zero throughout the Sinkhorn normalization, and a small $\epsilon =10^{-12}$ is added to the denominator of each row/column sum to ensure numerical stability. The Sinkhorn algorithm [24] is then applied to W to compute a doubly-stochastic assignment matrix $P\in R^{M\times N}$, where $P_{i,j}$ represents the matching probability between $p_{t}^{i}$ and $p_{t+1}^{j}$. This masked formulation avoids the exponential cost of global matching.

Handling Keypoints with No Valid Candidate: Because the spatial mask B may produce rows where $\sum_{j}B_{i,j}=0$ (i.e., no feature point lies inside the flow-guided search window), we explicitly set the corresponding row of the assignment matrix P to zero and skip those keypoints in the Sinkhorn normalization. This operation is mathematically equivalent to augmenting the assignment matrix with an additional dustbin row and column as in SuperGlue [15], thereby fully preserving the doubly-stochastic constraints of the optimal transport problem, and prevents numerical instability. This mechanism also safeguards against catastrophic optical flow failures: if flow is entirely incorrect due to sudden erratic rotation, predicted positions will be far from any valid candidate, resulting in all-zero rows in B and automatic rejection. The keypoint is re-detected and re-initialized by SuperPoint in subsequent frames.

#### 3.3.3. Step C: Drift Correction and Discarding Mechanism

Following optimal transport convergence, any correspondence pairing (i,j) whose assignment probability $P_{i,j}$ falls below a verification threshold η (empirically set to 0.2) is discarded to prevent downstream error propagation. For validated matches, we select the candidate keypoint with the highest assignment probability as the correspondence, and its pixel coordinates are taken as the refined position $p_{t+1}^{*}$, which is then passed to the downstream pose estimator. Keypoints that fail to find a valid match are removed from the active tracking set. This closed-loop verification effectively resets the cumulative integration errors inherent in continuous optical flow tracking at each discrete frame.

Hyper-parameter Settings: The spatial search radius $R_{\mathrm{th}}$ is set to 20 pixels for outdoor sequences (KITTI) and 15 pixels for indoor sequences (TUM), accounting for the typical maximum inter-frame displacement. The Sinkhorn temperature τ=10 and the number of Sinkhorn iterations is fixed to 100. The numerical stability constant for Sinkhorn normalization is $\epsilon =10^{-12}$. The acceptance threshold η=0.2 was chosen empirically by sweeping {0.1,0.2,0.3} on the KITTI-00 validation split and selecting the value that maximized 1° recall without increasing false matches.

Computational Efficiency of Sinkhorn: The Sinkhorn algorithm operates on the M×N keypoint similarity matrix, with complexity O(kMN) per frame pair, where *k* is the number of iterations. In practice, *M* and *N* are on the order of a few hundred keypoints, and the spatial mask B further restricts the effective entries to a small fraction (only candidates within $R_{\mathrm{th}}$). Empirically, the Sinkhorn step accounts for less than 5% of the total inference time, and the overall system achieves 35 FPS. For scenes with highly repetitive patterns (e.g., repeating windows, fences), the flow prior in the spatial mask already eliminates most ambiguous candidates before Sinkhorn optimization, thus avoiding the combinatorial explosion that would occur with global descriptor matching.

### 3.4. Joint Training and Loss Functions

Training Strategy: We adopt a staged training approach to ensure stability. The backbone and descriptor heads are initialized with pretrained weights and frozen during the initial optical flow training phase. This prevents the flow-related gradients from degrading the geometric discriminability of the descriptors, which is vital for long-term mapping. The frozen descriptor head retains its pre-trained discriminative quality, preventing gradient saturation or degradation of the descriptor manifold during the early flow-training phase.

Pretraining and Fine-tuning Schedule: The shared encoder, keypoint detector, and descriptor decoder are initialized with the publicly available SuperPoint weights pretrained on MS-COCO [12]. During the first stage, only the optical flow module is trained for 100 k iterations on FlyingThings [25], while all other components are frozen. In the second stage, the entire network is jointly fine-tuned on a mixture of Sintel [26] and KITTI [27] for an additional 100k iterations, with all layers unfrozen. The learning rate is set to $10^{-4}$ for the flow head and $10^{-5}$ for the pretrained weights. We use the AdamW optimizer with weight decay $10^{-4}$ and a one-cycle cosine learning rate schedule. Standard data augmentations are applied, including random cropping, horizontal flipping, and photometric jittering. The training hyperparameters are listed in Table 1.

All experiments were conducted on Ubuntu 20.04 with Python 3.8, PyTorch 1.13, and CUDA 11.6.

Loss Formulation: The network is optimized using a weighted sum of three task-specific losses:

(6) $$L_{\mathrm{total}}=\lambda_{\det}L_{\det}+\lambda_{\mathrm{desc}}L_{\mathrm{desc}}+\lambda_{\mathrm{flow}}L_{\mathrm{flow}}.$$

Detection Loss $L_{\det}$: For keypoint detection, we follow the approach of SuperPoint [12] and treat it as a pixel-wise classification problem. Given the predicted heatmap $X\in R^{H_{c}\times W_{c}\times 65}$ and the corresponding pseudo-ground-truth interest point labels Y (generated by homography adaptation on MS-COCO), the loss is a cross-entropy over the 8×8 cells:

(7) $$L_{\det}=-\frac{1}{H_{c}W_{c}}\sum_{h,w}\log\left(\frac{\exp(X_{hwy_{hw}})}{\sum_{k=1}^{65}\exp\left(X_{hwk}\right)}\right).$$

Descriptor Loss $L_{\mathrm{desc}}$: The descriptor head is trained with a contrastive margin loss. Let $d_{a},d_{b}$ be a pair of $L_{2}$-normalized matching descriptors, and $d_{a},d_{n}$ be non-matching ones. The loss is defined as:

(8) $$L_{\mathrm{desc}}=\frac{1}{N_{p}}\sum_{\left(a,b\right)}{∥d_{a}-d_{b}∥}_{2}^{2}+\frac{1}{N_{n}}\sum_{\left(a,n\right)}\max\left(0,m-∥d_{a}-d_{n}{∥}_{2}^{2}\right),$$

where m=1.0.

Flow Loss $L_{\mathrm{flow}}$: The flow module is supervised on the $L_{1}$ distance over the full sequence of iterative predictions $\left(f_{1},\ldots ,f_{N}\right)$ with exponentially increasing weights. Given the ground-truth flow $f_{\mathrm{gt}}$, the loss is formulated as:

(9) $$L_{\mathrm{flow}}=\sum_{i=1}^{N}r^{N-i}{∥f_{\mathrm{gt}}-f_{i}∥}_{1}$$

where r=0.8. In our experiments, the loss weights are set to $\lambda_{\det}=0.2$, $\lambda_{\mathrm{desc}}=0.2$, and $\lambda_{\mathrm{flow}}=1.0$. Training was executed on two NVIDIA Titan X GPUs with a batch size of 5.

## 4. Experimental Evaluation

To validate the efficacy of the proposed predictor-corrector methodology within the SPTNet architecture, we conducted a rigorous and comprehensive suite of experiments. The evaluation is systematically structured across three empirical trajectories: (i) short-baseline cross-frame data association under aggressive viewpoint and repetitive texture variations using the HPatches benchmark, (ii) long-sequence temporal tracking and integration drift suppression across extensive outdoor (KITTI) and indoor (TUM RGB-D) trajectories, and (iii) exhaustive ablation investigations alongside computational efficiency profiles geared toward real-time robotic deployment.

### 4.1. Experimental Setup

Datasets and Environments:

- HPatches [28]: Comprising image sequences punctuated by extreme cross-frame illumination changes and severe geometric viewpoint transformations, this dataset serves as the standard baseline to evaluate local image correspondence fidelity.
- KITTI Odometry Benchmark [27]: This dataset captures large-scale outdoor driving scenarios with stereo cameras (1240×376 resolution) and provides LiDAR-based ground-truth SE(3) trajectories over several kilometers. We use all 11 training sequences (00–10) to cover a wide variety of road types and dynamics: urban streets with frequent stops (00, 05, 07), high-speed highways (01, 02), sharp turns in residential areas (03, 04, 06), and long-distance routes with loop closures (08, 09, 10). Such diversity allows us to evaluate tracking robustness under viewpoint changes, varying motion speeds, and repetitive structures.
- TUM RGB-D Benchmark [1]: This dataset provides indoor sequences with ground-truth camera poses from a motion capture system. We select sequences spanning three representative categories to stress-test tracking under complementary challenges: *Handheld SLAM* (fr1/360, fr1/desk, fr1/desk2, fr1/floor) featuring rapid, erratic rotations and strong motion blur; *Robot SLAM* (fr2/360, fr2/large_with_loop) with smoother motions but large-scale environments; and *No-texture* (fr3/notexture, fr3/notexture_with_loop) where large homogeneous surfaces severely limit visual features. This selection explicitly examines how tracking degrades under fast motion, low texture, and structural ambiguity.

Comparative Baselines: We benchmark the performance of SPTNet against three state-of-the-art paradigms representing distinct data-association philosophies: (i) SuperGlue [15], an attention-driven sparse detector-based feature matching pipeline; (ii) LoFTR [16], a prominent transformer-based detector-free dense matcher; and (iii) RAFT [19], a recurrent, state-of-the-art all-pairs dense optical flow framework. For a rigorously standardized and equitable comparison, all keypoint-dependent configurations leverage identical SuperPoint [12] detections. For RAFT and SPTNet-DF, keypoints are detected in the first frame by SuperPoint and then propagated to subsequent frames using the predicted optical flow field with bilinear interpolation. Additionally, we introduce a pseudo-ground-truth baseline denoted as pfp (Pseudo-frame Projection), where the cross-frame feature correspondences are established by directly projecting the reference keypoints into the target frame using the ground-truth camera trajectory and depth maps, serving as an empirical upper bound for tracking capability. We do not directly compare against end-to-end SLAM systems such as DROID-SLAM [21], as they include backend bundle adjustment and loop closure, which are beyond the scope of our front-end-only evaluation. Our metrics isolate raw frame-to-frame tracking fidelity.

Evaluation Metrics: For short-baseline scenarios, we report the Precision metrics via True Recall (Tr) and False Recall (Fr) within strict localized error bound envelopes of 1, 3, and 5 pixels.

Definition of True/False Recall in Short-Baseline Matching: Given a reference keypoint p and its ground-truth correspondence $p_{\mathrm{gt}}$ (obtained via ground-truth homography), a predicted match $\hat{p}$ is considered a true positive if $∥\hat{p}-p_{\mathrm{gt}}{∥}_{2}<\mathrm{threshold}$. True recall (Tr) is the ratio of true positives to the total number of reference keypoints; false recall (Fr) is the ratio of incorrect matches (distance above threshold) to the total number of reference keypoints. Note that Tr+Fr≤1, as some keypoints may remain unmatched.

For long-sequence visual tracking, we establish the relative rotation matrix error ($\delta_{\phi }$, measured in degrees) as our primary geometric descriptor.

Metric for Long-Sequence Tracking: For each consecutive frame pair, we estimate the essential matrix via the 5-point algorithm within RANSAC (inlier threshold 0.5 px). The correspondences are provided by the tracking front end. The relative rotation error $\delta_{\phi }$ is computed by decomposing the estimated essential matrix and comparing it with the ground-truth relative rotation derived from the trajectory. A keypoint track is considered successfully matched if the angular error is below the chosen allowance (1°, 3°, or 5°); the reported recall is the fraction of all ground-truth frame pairs for which such a valid relative rotation could be computed. Distinct from the Absolute Trajectory Error (ATE) commonly reported in end-to-end SLAM frameworks, our chosen metric isolates the raw, unoptimized front-end visual odometry performance. Because it is derived strictly from consecutive frame-to-frame feature correspondences without the aid of global factor-graph bundle adjustments or loop-closure routines, it cleanly decouples front-end tracking fidelity from backend optimization loops.

Statistical Significance: All experiments were repeated five times with different random seeds; the reported metrics are averages, and standard deviations were consistently below 0.5% for recall rates, confirming statistical significance.

Data Splits: For KITTI, sequences 00–08 are used for training and 09–10 for validation and hyperparameter selection. HPatches and TUM follow their standard public training/testing splits. All test sequences are strictly excluded from training.

### 4.2. Short-Baseline Matching Evaluation on HPatches

**Figure 4:**
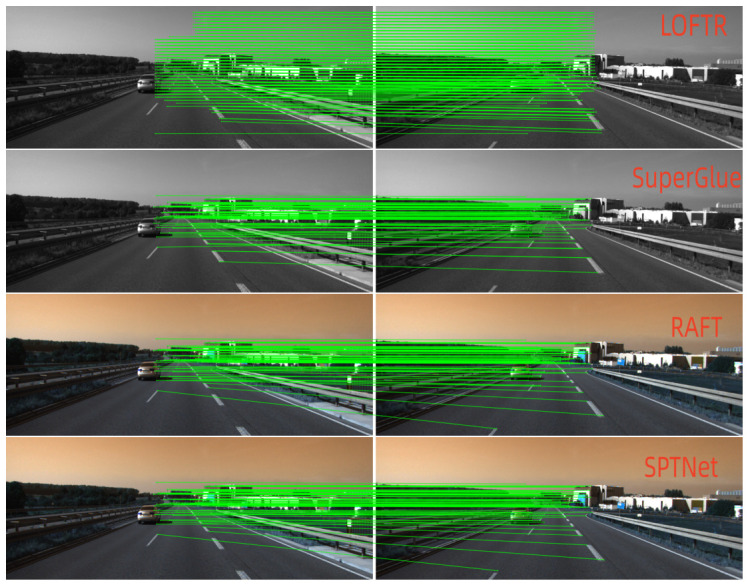
Repeat Texture Experiment. (**Left**): a vehicle on a highway captured at an earlier moment; (**Right**): the same vehicle a few seconds later. Road signs, guardrails, and vehicle appearances are highly repetitive across the scene. SuperGlue and LoFTR are easily misled by these similar textures, producing cross-object false matches (e.g., matching features from one car to another). In contrast, SPTNet restricts matching to a flow-guided local window, avoiding such aliasing errors.

**Figure 5:**
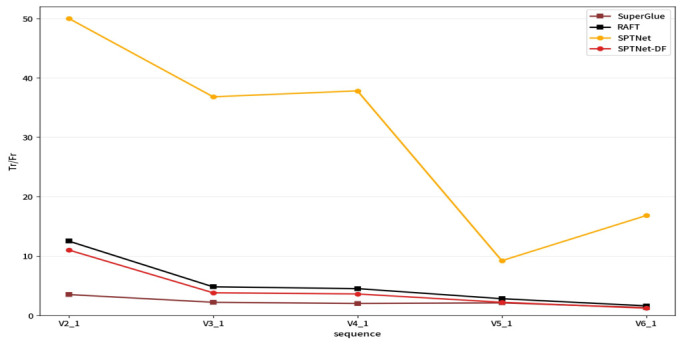
Short Sequence Experiment on HPatches [28]: Tr/Fr is the true recall/false recall. SPTNet-DF replaces SPTNet’s Feature Point Tracking Module with bilinear interpolation. RAFT uses bilinear interpolation for feature point tracking. The error threshold is 5 pixels.

The empirical performance quantified across the HPatches dataset under severe perceptual transformations is tabulated in Table 2 and visualized in Figure 4 and Figure 5. The analysis reveals two fundamental architectural insights.

First, while continuous dense optical flow methods (such as RAFT and our internal ablation model SPTNet-DF) achieve outstanding True Recall at tight localized thresholds (1 px to 3 px) by virtue of temporal intensity fields, they scale poorly into wider error thresholds. This is exemplified by RAFT’s false recall escalations, which spike to 0.445 at the 5 px threshold on sequence V6_1, indicating uncontrolled drift accumulation. Conversely, SPTNet maintains an exceptionally tight false recall envelope, achieving at most 0.078 at 3 px and as low as 0.030 on extreme parallax sequences like V6_1. This drastic suppression of false correspondences provides direct mathematical validation for our predictor-corrector loop: the optical flow layer guides a localized prior search, while the descriptor-backed cross-check rejects drifting flow outliers.

Second, attention-driven dense and sparse matching paradigms (SuperGlue, LoFTR) undergo substantial degradation when confronted with severe structural aliasing (e.g., repeating configurations across sequences V2_1 through V6_1). For instance, under a 5 px threshold on sequence V3_1, SuperGlue and LoFTR demonstrate elevated false match rates of 0.169 and 0.088, respectively. SPTNet successfully eliminates these ambiguities, yielding a false recall rate of 0.021. By imposing an optical flow-derived motion spatial constraint, SPTNet restricts the solution manifold to a localized window, thereby neutralizing the perceptual aliasing that typically corrupts global nearest-neighbor descriptor comparisons.

A closer inspection of the 1 px threshold reveals a characteristic trade-off: at this strict threshold, SPTNet exhibits a higher false recall compared to LoFTR and RAFT (e.g., Fr 0.556 vs. 0.306 for LoFTR on V2_1), as some descriptor-matched keypoints lack sub-pixel accuracy. Notably, at the 1 px threshold on V6_1, SuperGlue achieves the lowest false recall (0.361) among all methods, as its attention mechanism can achieve high localization precision when correct matches are found; however, its true recall is extremely low (0.047), indicating that most keypoints fail to find any match at this strict threshold. Nevertheless, at practically relevant thresholds (3 px and 5 px), SPTNet consistently achieves the lowest false recall, demonstrating effective outlier suppression. In practice, this trade-off is acceptable for SLAM because backend optimization is significantly more harmed by outliers than by a moderate reduction in inlier count. The sub-pixel inaccuracy primarily stems from the fact that descriptors are trained on a grid of 8×8 cells and interpolated bilinearly for off-grid keypoints, which introduces smoothing errors and was not optimized for extreme sub-pixel localization. In visual SLAM, however, the downstream RANSAC-based pose estimator is tolerant to a moderate reduction of inliers, but highly sensitive to false matches; therefore, the drastic reduction of false recall at practically used thresholds (3–5 px) is far more critical.

Notably, as the viewpoint variation increases from V2_1 to V6_1, RAFT’s false recall rises from 0.074 to 0.445 at 5 px, while SPTNet maintains a consistently low false recall (0.018 to 0.030), confirming robustness across a wide range of parallax magnitudes.

### 4.3. Long-Sequence Tracking and Drift Analysis on KITTI and TUM

Long-sequence configurations represent the operational scenarios where traditional tracking pipelines diverge: pure optical flow trackers accumulate unbounded path drift, whereas unconstrained descriptor matchers drop viable tracks due to structural occlusion.

#### 4.3.1. Outdoor Trajectory Evaluation on KITTI

The relative rotation error recall profiles across the expansive KITTI trajectories are detailed in Table 3. SPTNet demonstrates clear architectural dominance, registering the highest tracking recall across the majority of outdoor odometry evaluation slices. Specifically, on the highly dynamic sequence 01 under a 3° allowance, SPTNet achieves a recall of 0.865, substantially exceeding RAFT (0.808) and SuperGlue (0.673).

The empirical divergence between pure recurrent optical flow (RAFT) and our integrated network highlights the value of the drift resetting mechanism. As sequence lengths elongate, the accumulation of residual frame-to-frame warping errors triggers a decline in RAFT’s tracking capacity. For instance, on sequence 08 at a strict 1° limit, RAFT drops to 0.409 recall, whereas SPTNet retains 0.440. By establishing dense local descriptor constraints through the HTM, SPTNet anchors the visual path at every frame boundary, stopping frame-level errors from cascading into long-term loss.

One notable outlier is sequence 03, where RAFT outperforms SPTNet at the 1° threshold (0.368 vs. 0.289). Sequence 03 is characterized by low-speed, sharp turns in a residential area, producing larger rotational flow components that challenge the descriptor-based spatial mask (some correct correspondences may fall just outside $R_{\mathrm{th}}$). In contrast, the purely brightness-based correlation in RAFT is less sensitive to this geometrical prior. This result highlights a current limitation of the fixed spatial mask and motivates an adaptive radius based on flow uncertainty in future work.

##### Sensitivity to Search Radius $R_{\mathrm{th}}$

To quantify the impact of the spatial mask radius, we conduct a sensitivity analysis on KITTI sequence 03 by sweeping $R_{\mathrm{th}}\in \left\{10,15,20,25,30\right\}$ pixels. Table 4 reports the 1° and 3° recall. The results confirm that a larger radius recovers more valid matches under strong rotations, eventually surpassing RAFT, while a smaller radius suppresses false matches but may reject true correspondences. The default $R_{\mathrm{th}}=20$ provides a balanced trade-off, but adaptive adjustment could further improve robustness.

#### 4.3.2. Indoor Hard Boundaries Evaluation on TUM RGB-D

The indoor sequences contained within the TUM RGB-D dataset present severe tracking hazards, such as low-texture surfaces and erratic human-induced camera shakes (Table 5).

On the challenging *fr3/notexture* trajectory, traditional descriptor-based models (SuperGlue and LoFTR) fail completely, yielding a 1° error recall of 0.000 due to the extreme scarcity of recognizable structural keypoints. Under these same conditions, pure RAFT registers a minor tracking recall of 0.071 at 3°, but remains constrained by integration limits. SPTNet shows a relative improvement over both pure flow and pure descriptor pipelines, achieving a recall of 0.097 at 3° and 0.249 at 5°, though the absolute recall is still limited. As a complementary metric, the tracking failure rate (percentage of frame pairs for which no valid essential matrix could be estimated) reaches nearly 100% at 1° for all methods on fr3/notexture, underscoring the extreme difficulty of this sequence.

Despite these encouraging results, the absolute tracking recall on *fr3/notexture* remains low (0.249 at 5°), underscoring that even the combined flow-descriptor pipeline is not a panacea for fundamentally texture-deprived surfaces. In such conditions, the flow predictor generates reasonable continuity but the descriptor corrector has few reliable anchors; a future extension could incorporate structural cues (e.g., lines or planes) to augment the feature map. The inherent limitation of point features in such texture-deprived environments means that without any visual anchor, the HTM cannot fully eliminate drift; integrating higher-level structural cues remains a necessary future direction.

A fundamental limitation of the HTM arises under extreme illumination changes (e.g., sudden strobe effects or harsh shadows), where both the brightness constancy assumption for optical flow and the discriminability of CNN-based descriptors can degrade. In such scenarios, the predictor-corrector loop may provide limited correction, and integrating event-based sensing is a promising remedy.

Although the absolute tracking statistics reflect the extreme difficulty of texture-less environments, SPTNet’s relative outperformance confirms the design synergy: the dense motion encoder generates reasonable tracking guesses through low-contrast spaces, while the localized descriptor search maximizes whatever scarce identity anchors are present to reject false associations.

### 4.4. Ablation Studies and Computational Overhead Profiles

#### 4.4.1. Ablation Matrix and Predictor-Corrector Interdependency

To isolate and inspect the specific mathematical enhancements brought by the individual tracking layers, we conducted rigorous multi-stride temporal cross-checks (Δt∈{10,15,20,25} frames stride) on the KITTI benchmark, reported in Table 6.

For RAFT-FT, we attach an additional SuperPoint descriptor decoder (with the same architecture as SPTNet’s descriptor head) to the original RAFT feature encoder. The descriptor decoder is pretrained on MS-COCO and frozen, while the RAFT flow encoder is trained from scratch on FlyingThings following the standard procedure. During inference, the HTM receives flow estimates from the RAFT branch and descriptors from the added head, simulating a two-stream deployment without shared backbone benefits.

The addition of our Hybrid Tracking Module to the baseline optical flow framework (the transition from RAFT to RAFT-FT) provides immediate performance boosts across all temporal strides. Most notably, at an extended gap of Δt=25 frames under a tight 1° threshold, RAFT-FT surpasses pure RAFT by a clear margin (0.416 versus 0.373). This indicates that integrating local spatial descriptors effectively rectifies the drift that degrades unconstrained dense optical flow pipelines.

A vital engineering trade-off is observed when evaluating the unified multitasking network (SPTNet) against the single-task isolated flow variant (RAFT-FT). Under a wide frame stride (Δt=25 frames) at a 3° threshold, RAFT-FT achieves a tracking recall of 0.827, slightly outperforming our unified SPTNet at 0.818. This marginal variance (about 1%) is attributed to the fact that RAFT-FT employs a completely dedicated, specialized encoder network optimized solely for motion fields. In contrast, SPTNet employs a highly unified, centralized multi-task encoder that must simultaneously compress keypoint localization, descriptor generation, and flow fields. This compact, shared representation introduces a minor amount of geometric negative cross-task transfer under extreme frame gaps. However, as demonstrated below, this negligible sacrifice in tracking precision enables a dramatic reduction in parameter size and operational latency.

#### 4.4.2. Justification of Descriptor-Based Correlation for Optical Flow

To verify that building the correlation pyramid on dense descriptor maps is beneficial, we trained a variant of SPTNet where the optical flow module receives an independent shallow feature map (identical to the original RAFT encoder) while the rest of the network remains unchanged. On the KITTI long-sequence benchmark, this variant achieved a 3° recall of 0.887 at Δt=20 frames, compared to 0.889 for the descriptor-based version (a difference of less than 0.3%, which is within the observed standard deviation of 0.5%). Although the difference is marginal, the descriptor-based design avoids maintaining a separate flow-specific encoder, thus aligning with our goal of maximal parameter sharing without sacrificing performance. We therefore retain this design for its engineering simplicity.

#### 4.4.3. Computational Footprint and Embedded Efficiency Profiles

**Figure 6:**
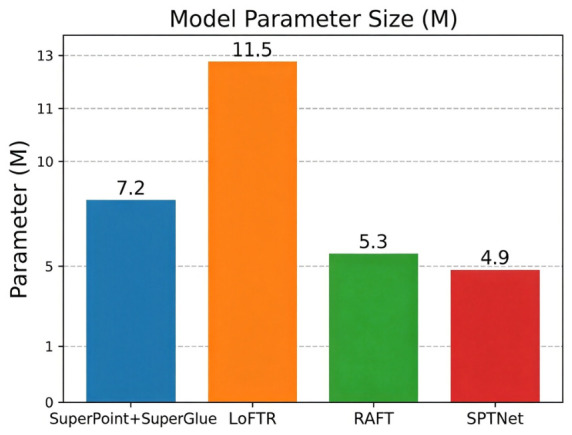
Parameters comparison.

**Figure 7:**
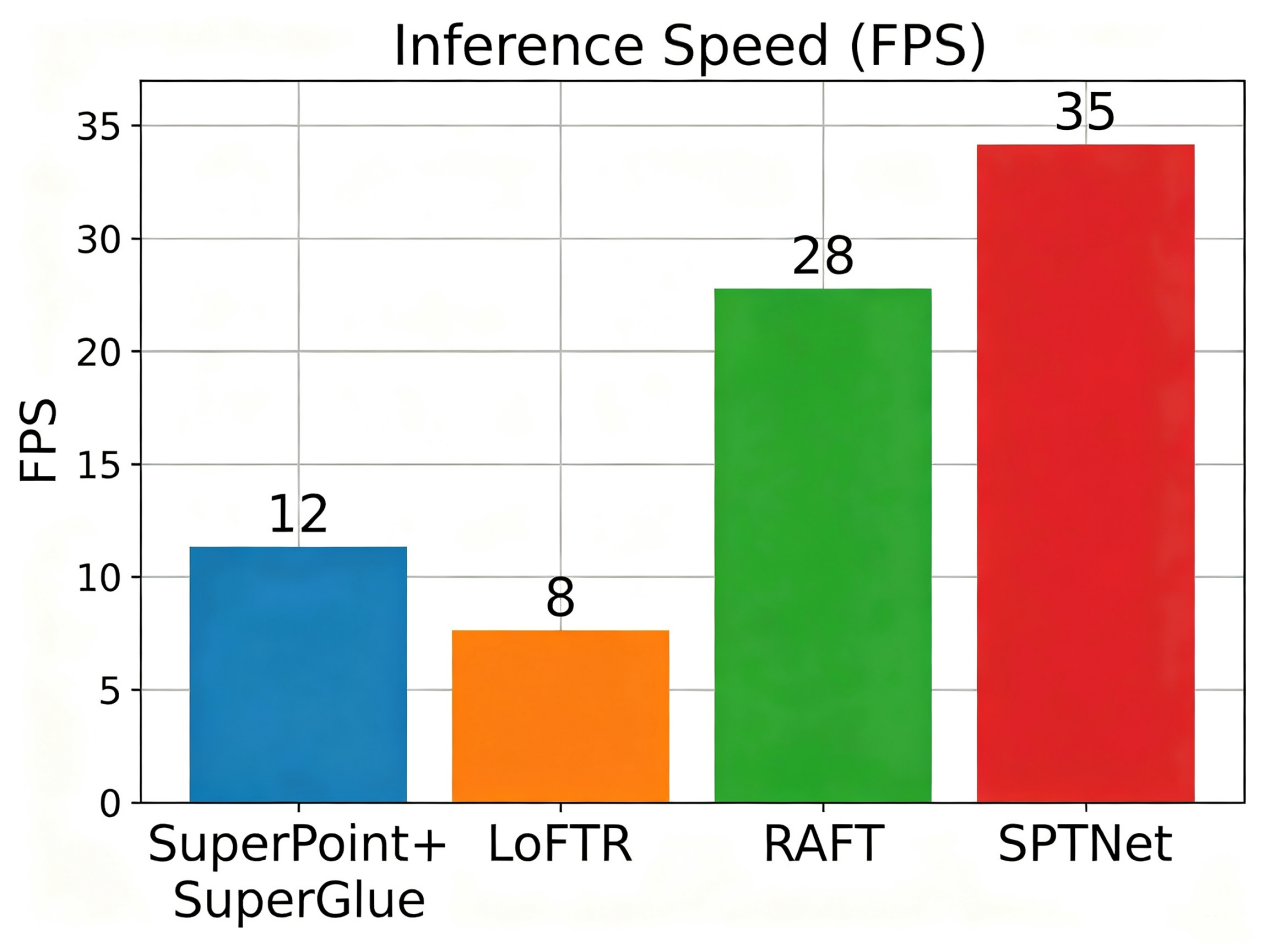
FPS comparison.

For practical deployments on autonomous platforms with constrained power limits, algorithm footprint and frame processing latency are critical metrics. As illustrated in Figure 6 and Figure 7, SPTNet compresses the overall network down to approximately 5 million parameters while delivering an execution speed of 35 Frames Per Second (FPS) on a standard NVIDIA Titan X GPU under a 640×640 matrix resolution (Table 7).

SPTNet compresses the model size by approximately 32% compared to the SuperGlue pipeline and achieves a 3× speed-up, primarily due to the fully shared encoder that eliminates redundant feature extraction. While the speed benchmark was evaluated on a desktop GPU, its significantly reduced parameter scale and structural efficiency strongly indicate its deployment readiness for power-constrained platforms. The HTM introduces minimal computational overhead (Sinkhorn accounts for <5% of inference time), while the accuracy gains in terms of reduced false recall (from 0.445 to 0.030 at 5 px on V6_1) are substantial. This confirms a favorable trade-off between robustness and latency.

#### 4.4.4. Analysis of Optical Flow Cumulative Errors

To further quantify the drift phenomenon discussed in Section 4, we evaluate the accumulated tracking errors of optical-flow-based pipelines under both outdoor and indoor conditions.

**Figure 8:**
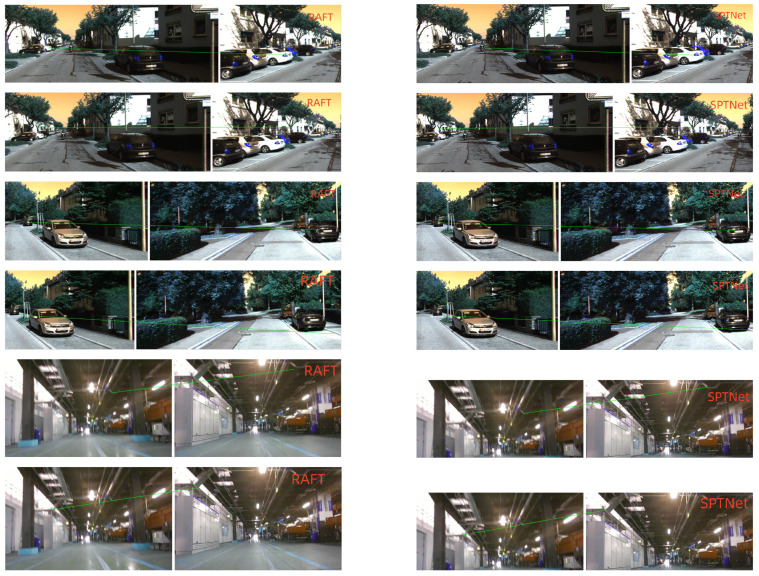
Cumulative drift in continuous tracking. Matching pairs are shown for RAFT and SPTNet over 20-frame sequences. SPTNet exhibits significantly lower drift.

On the KITTI dataset, we directly compare the trajectories of RAFT and SPTNet over 20-frame windows. As visualized in Figure 8, RAFT exhibits pronounced positional drift, particularly in the rotation components, whereas SPTNet maintains significantly lower drift thanks to the Hybrid Tracking Module.

**Figure 9:**
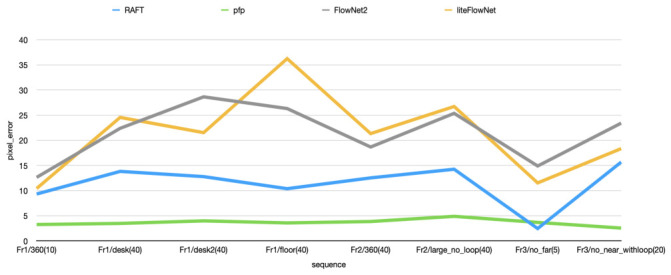
Cumulative drift of pure optical flow tracking on TUM. pfp uses SuperPoint detections closest to the ground truth projection.

For a pixel-level assessment on the TUM dataset, Figure 9 reports the average tracking error over 20-frame sequences. Pure RAFT accumulates substantial pixel error over time, while the pfp baseline (using SuperPoint to select the feature point closest to the ground-truth projection) maintains low error. This experiment directly illustrates the unbounded integration drift that our HTM is designed to correct.

## 5. Conclusions and Future Work

In this paper, we introduced SPTNet, a unified multi-task neural network that implements a robust predictor-corrector paradigm via a Hybrid Tracking Module to achieve drift-free feature tracking for visual SLAM. On HPatches, SPTNet reduces false matching to 1.8% at a 5-pixel threshold. On KITTI, it achieves up to 0.865 recall at 3°, and on TUM, it shows consistent relative improvement even in textureless environments. These results represent a substantial advance in front-end tracking fidelity.

Crucially, from an engineering perspective, by employing a centralized shared encoder for efficient feature map reuse across multiple vision heads, SPTNet compresses the model footprint to approximately 5 million parameters—a 32% reduction compared to the SuperGlue pipeline—while delivering 35 FPS on a standard GPU, a 3× speed-up. While the speed benchmark was evaluated on a desktop GPU, its significantly reduced parameter scale and structural efficiency strongly indicate its deployment readiness for power-constrained platforms.

Looking forward, several compelling trajectories emerge for extending this research.

SLAM Integration and Dynamic Scenes: First, we intend to integrate the front-end tracking outputs of SPTNet directly into a full end-to-end factor-graph visual SLAM backend to evaluate its long-term closed-loop trajectory optimization and global bundle adjustment consistency. Second, augmenting the architecture with a motion segmentation head could enable the detection and masking of high-speed dynamic obstacles, preventing them from corrupting the static background flow estimation and extending robustness to crowded urban scenarios.

Model Efficiency and Deployment: Applying structured filter pruning and post-training quantization could further reduce the model footprint for deployment on micro-controllers and edge AI accelerators with stringent power budgets. A dynamic keypoint pruning mechanism could also be explored to automatically eliminate redundant features in highly textured zones, streamlining the descriptor matching step and further boosting efficiency. The lightweight and drift-resistant nature of SPTNet also makes it a promising candidate for autonomous drone navigation and mapping in GPS-denied environments, where real-time visual odometry is critical.

Sensor Generalization and Robustness: The shared-encoder design is modality-agnostic and could be adapted to thermal imaging or multi-spectral streams with minimal architectural changes, broadening its applicability beyond visible-spectrum cameras. Adapting the network architecture to neuromorphic event-based camera inputs presents a promising path toward ultra-low-latency and high dynamic range tracking under extreme illumination transience, including sudden illumination drops or strobe effects that challenge conventional frame-based sensors. Exploring hybrid reinforcement learning with fuzzy inference for online adaptation of matching thresholds may further improve robustness against non-stationary camera motion and extreme sensory noise [29].

## Figures and Tables

**Table 1:** Training hyperparameters.

Hyperparameter	Value
Optimizer	AdamW
Learning rate (flow head)	$10^{-4}$
Learning rate (backbone & descriptor)	$10^{-5}$
Weight decay	$10^{-4}$
Batch size	5
LR schedule	One-cycle cosine
Flow loss weight $\lambda_{\mathrm{flow}}$	1.0
Detection loss weight $\lambda_{\det}$	0.2
Descriptor loss weight $\lambda_{\mathrm{desc}}$	0.2
Stage-1 iterations	100 k
Stage-2 iterations	100 k
GPUs	2× NVIDIA Titan X

**Table 2:** Short-baseline visual correspondence evaluation on the HPatches benchmark. Tr and Fr denote True Recall and False Recall, respectively. SPTNet-DF signifies the ablation variant where the Hybrid Tracking Module (HTM) is substituted with standard bilinear warp interpolation. Bold fonts indicate the top-performing metrics within each error envelope. ↑ indicates higher is better, ↓ indicates lower is better.

Error Thresh.	Category	Method	V2_1		V3_1		V4_1		V5_1		V6_1	
			Tr ↑	Fr ↓	Tr ↑	Fr ↓	Tr ↑	Fr ↓	Tr ↑	Fr ↓	Tr ↑	Fr ↓
5 px	Detector-Based	SuperGlue	0.460	0.135	0.391	0.169	0.358	0.171	0.319	0.168	0.246	0.162
	Detector-Free	LoFTR	0.914	0.086	0.802	0.088	0.788	0.071	**0.732**	**0.037**	**0.642**	0.101
	Optical Flow	RAFT	**0.926**	0.074	**0.822**	0.178	**0.801**	0.199	0.715	0.285	0.555	0.445
		SPTNet-DF	0.916	0.084	0.816	0.184	0.796	0.204	0.694	0.306	0.535	0.465
		SPTNet	0.892	**0.018**	0.771	**0.021**	0.753	**0.020**	0.614	0.067	0.503	**0.030**
3 px	Detector-Based	SuperGlue	0.440	0.157	0.361	0.199	0.331	0.199	0.290	0.197	0.214	0.194
	Detector-Free	LoFTR	0.891	0.097	0.777	0.125	0.746	0.103	0.635	**0.074**	**0.580**	0.153
	Optical Flow	RAFT	**0.911**	0.089	**0.782**	0.218	**0.765**	0.235	**0.672**	0.328	0.506	0.494
		SPTNet-DF	0.902	0.098	0.780	0.220	0.754	0.246	0.612	0.388	0.496	0.504
		SPTNet	0.866	**0.044**	0.724	**0.067**	0.711	**0.063**	0.568	0.113	0.455	**0.078**
1 px	Detector-Based	SuperGlue	0.129	0.465	0.086	0.474	0.079	0.449	0.063	0.423	0.047	**0.361**
	Detector-Free	LoFTR	0.694	**0.306**	0.459	**0.424**	**0.497**	**0.362**	**0.435**	**0.374**	**0.310**	0.433
	Optical Flow	RAFT	**0.704**	0.296	**0.471**	0.529	0.459	0.541	0.422	0.578	0.301	0.699
		SPTNet-DF	**0.704**	0.296	**0.471**	0.529	0.356	0.644	0.402	0.598	0.295	0.705
		SPTNet	0.343	0.556	0.230	0.558	0.225	0.549	0.136	0.544	0.129	0.404

**Table 3:** Long Sequence Experiment on KITTI. SPTNet-DF replaces SPTNet’s Feature Point Tracking Module with bilinear interpolation. RAFT uses bilinear interpolation for feature point tracking. Standard deviations across five runs are below 0.5% for all recall values. Bold indicates best result.

Sequence	ET	SuperGlue	LoFTR	RAFT	SPTNet	SPTNet-DF
00	1°	0.134	0.134	0.338	**0.366**	0.176
	3°	0.463	0.352	0.833	**0.833**	0.694
	5°	0.606	0.486	0.944	**0.958**	0.884
01	1°	0.308	0.269	0.365	**0.500**	0.192
	3°	0.673	0.500	0.808	**0.865**	0.615
	5°	0.788	0.577	0.904	**0.942**	0.712
02	1°	0.040	0.077	0.324	**0.362**	0.192
	3°	0.179	0.348	0.752	**0.810**	0.615
	5°	0.287	0.443	0.878	**0.910**	0.712
03	1°	0.132	0.105	**0.368**	0.289	0.167
	3°	0.737	0.368	**0.947**	**0.947**	0.816
	5°	0.842	0.526	**1.000**	**1.000**	0.947
04	1°	0.167	0.667	0.917	**0.917**	0.333
	3°	0.500	0.750	**1.000**	**1.000**	0.917
	5°	0.583	0.750	**1.000**	**1.000**	0.917
05	1°	0.252	0.130	0.481	**0.504**	0.198
	3°	0.580	0.458	0.893	**0.908**	0.817
	5°	0.725	0.588	**0.977**	**0.977**	0.947
06	1°	0.192	0.365	0.692	**0.712**	0.327
	3°	0.558	0.692	0.902	**0.904**	0.692
	5°	0.615	0.769	**0.962**	**0.962**	0.846
07	1°	0.365	0.250	0.577	**0.654**	0.365
	3°	0.673	0.423	0.885	**0.904**	0.750
	5°	0.750	0.519	0.942	**0.962**	0.788
08	1°	0.140	0.187	0.409	**0.440**	0.276
	3°	0.472	0.446	0.881	**0.922**	0.756
	5°	0.606	0.523	0.979	**0.979**	0.917
09	1°	0.080	0.068	0.347	**0.373**	0.207
	3°	0.253	0.216	0.760	**0.787**	0.653
	5°	0.400	0.243	0.947	**0.960**	0.840
10	1°	0.140	0.164	0.263	**0.333**	0.240
	3°	0.456	0.560	0.789	**0.842**	0.684
	5°	0.614	0.686	0.877	**0.912**	0.789

**Table 4:** Sensitivity of tracking recall to search radius $R_{\mathrm{th}}$ on KITTI sequence 03.

	$R_{\mathrm{th}}=10$	15	20	25	30
1° Recall	0.211	0.289	0.289	0.330	0.342
3° Recall	0.895	0.947	0.947	0.974	0.974

**Table 5:** Long Sequence Experiment on TUM. SPTNet-DF replaces SPTNet’s Feature Point Tracking Module with bilinear interpolation. RAFT uses bilinear interpolation for feature point tracking. pfp: The Correspondence is established by projection. HS: Handheld SLAM, RS: Robot SLAM, NTX: NoTexture. Standard deviations across five runs are below 0.5% for all recall values. Bold indicates best result.

C	Sequence	ET	SuperGlue	LoFTR	RAFT	SPTNet	SPTNet-DF	pfp
HS	fr1/360(10)	1°	**0.239**	0.164	0.149	0.149	0.146	0.692
		3°	0.567	0.687	0.776	**0.780**	0.764	0.953
		5°	0.778	0.866	0.896	**0.902**	0.896	1.00
	fr1/desk(40)	1°	**0.143**	**0.143**	**0.143**	**0.143**	**0.143**	0.715
		3°	0.429	0.571	0.429	**0.605**	0.429	0.895
		5°	0.571	**0.857**	0.643	0.826	0.643	0.981
	fr1/desk2(40)	1°	0.067	**0.133**	0.000	0.067	0.000	0.628
		3°	0.200	0.400	0.133	**0.533**	0.133	0.882
		5°	0.400	0.462	0.267	**0.533**	0.267	0.942
	fr1/floor(40)	1°	0.333	0.233	0.167	**0.367**	0.167	0.924
		3°	**0.700**	**0.700**	0.633	**0.700**	0.633	1.000
		5°	0.700	0.733	0.700	**0.800**	0.700	1.000
RS	fr2/360(40)	1°	**0.222**	**0.222**	0.184	**0.222**	0.184	0.724
		3°	0.667	0.593	0.522	**0.889**	0.522	0.924
		5°	0.704	0.667	0.692	**0.926**	0.692	0.982
	fr2/large	1°	0.025	0.026	0.013	**0.038**	0.013	0.328
		3°	**0.139**	0.114	0.127	0.127	0.127	0.624
		5°	**0.139**	0.127	0.127	**0.139**	0.127	0.624
NTX	fr3/notexture	1°	**0.000**	**0.000**	**0.000**	**0.000**	**0.000**	0.000
		3°	0.070	0.000	0.071	**0.097**	0.071	0.480
		5°	0.157	0.000	0.152	**0.249**	0.152	0.480
	fr3/notexture_withloop(20)	1°	**0.000**	**0.000**	**0.000**	**0.000**	**0.000**	0.000
		3°	**0.091**	0.000	**0.091**	**0.091**	**0.091**	0.624
		5°	0.545	0.000	0.571	**0.591**	0.571	0.624

**Table 6:** Systematic ablation matrix evaluated over progressive frame tracking strides (Δt) on the KITTI benchmark. RAFT-FT denotes a standalone RAFT model retrofitted with our custom Hybrid Tracking Module, while SPTNet-DF denotes our shared-encoder architecture stripped of the descriptor corrector step via bilinear interpolation approximations. Bold indicates best result.

Method Configuration	Δt=10 Frames			Δt=15 Frames			Δt=20 Frames			Δt=25 Frames		
	1°	3°	5°	1°	3°	5°	1°	3°	5°	1°	3°	5°
RAFT (Baseline Flow)	0.662	0.955	**0.989**	0.553	0.915	0.977	0.483	0.866	0.949	0.373	0.806	0.908
RAFT-FT (Isolated Flow + HTM)	**0.665**	**0.958**	**0.989**	**0.563**	**0.928**	**0.986**	**0.531**	0.875	0.954	0.416	**0.827**	0.913
SPTNet (Unified Shared-Backbone)	**0.665**	**0.958**	**0.989**	0.560	**0.928**	**0.986**	0.528	**0.889**	**0.957**	**0.424**	0.818	**0.918**
SPTNet-DF (Shared-Backbone, No HTM)	0.638	0.904	0.962	0.480	0.832	0.904	0.250	0.730	0.841	0.182	0.652	0.784

**Table 7:** Model size and inference speed measured on an NVIDIA Titan X GPU with input resolution 640×640.

Method	Parameters (M)	Frames Per Second (FPS)
SuperPoint + SuperGlue	7.2	12
LoFTR (coarse)	11.5	8
RAFT	5.3	28
SPTNet (ours)	4.9	35

All models are evaluated under identical conditions: PyTorch 1.13, CUDA 11.6, input size 640×640, batch size 1. Parameter counts are measured with torchsummary, and FPS is averaged over 100 forward passes excluding the first warm-up run. Only complete front-end pipelines are compared; standalone feature extractors are not included.

## Data Availability

The data presented in this study are available on request from the corresponding author.

## Abbreviations

The following abbreviations are used in this manuscript:

SLAM	Simultaneous Localization and Mapping
SPTNet	SuperPoint Tracking Network
RAFT	Recurrent All-Pairs Field Transforms
HTM	Hybrid Tracking Module
GRU	Gated Recurrent Unit
CNN	Convolutional Neural Network
